# ﻿Morphological and molecular analyses reveal two new species of *Grifola* (Polyporales) from Yunnan, China

**DOI:** 10.3897/mycokeys.102.118518

**Published:** 2024-02-29

**Authors:** Song-Ming Tang, De-Chao Chen, Shuai Wang, Xiao-Qu Wu, Cheng-Ce Ao, Er-Xian Li, Hong-Mei Luo, Shu-Hong Li

**Affiliations:** 1 Biotechnology and Germplasm Resources Institute, Yunnan Academy of Agricultural Sciences, Kunming 650205, China Biotechnology and Germplasm Resources Institute, Yunnan Academy of Agricultural Sciences Kunming China; 2 School of Agriculture, Yunnan University, Kunming 650504, China Yunnan University Kunming China; 3 College of Agriculture and Biological Science, Dali University, Dali 671003, China Dali University Dali China

**Keywords:** 2 new species, morphology, multi-gene phylogeny, Southeast Asia, taxonomy, Yunnan

## Abstract

Species of *Grifola* are famous edible mushrooms and are deeply loved by consumers around the world. Most species of this genus have been described and recorded in Oceania, Europe and South America, with only *Grifolafrondosa* being recorded in Asia. In this study, two novel species of *Grifola* from southwestern China (Asia) are introduced. Macro and micromorphological characters are described. *Grifolaedulis***sp. nov.** present medium-size basidiomata with gray to gray-brown lobes upper surface, mostly tibiiform or narrowly clavate, rarely narrowly lageniform or ellipsoid chlamydospores, cuticle hyphae terminal segments slightly enlarged. *Grifolasinensis***sp. nov.** has white to grayish white lobes upper surface, mostly ellipsoid, rarely narrowly utriform chlamydospores, and broadly ellipsoid to ellipsoid basidiospores (4.6–7.9 × 3.0–5.9 μm). The two new species are supported by phylogenetic analyses of combined nuclear rDNA internal transcribed spacer ITS1-5.8S-ITS2 rDNA (ITS) and β-tubulin (*TUBB*). Moreover, the genetic distance between *TUBB* sequences of those specimen from GenBank was 1.76–1.9%. Thus, the conspecificity relationship of our specimens remains uncertain, and further specimens are required to conclusively confirm its identity.

## ﻿Introduction

*Grifola*Gray (1821) was established based on the type species, *G.frondosa* (Dicks.) Gray (Gray 1821). *Grifola* species are characterized by their compound basidiomata developing on the ground from roots at the base of trees or stumps and causing white-rot (Gray 1821; Rajchenberg 2002; Ryvarden and Melo 2014). The genus presents monomitic or dimitic hyphal system with clamped generative hyphae, basidiospores ovoid to ellipsoid, inamyloid, and abundant chlamydospores in culture (Rugolo et al. 2023).

To date, six species of *Grifola* have been described worldwide, of which two reported from North Hemisphere (*G.frondosa* (Dicks.) Gray is widely distributed in Asia, North America and Europe (Rajchenberg 2002) and *G.amazonica* Ryvarden is only known from Brazil (Ryvarden 2004)), four species reported from South Hemisphere (*G.sordulenta* (Mont.) Singer from Argentina, Chile, New Zealand and Patagonia, *G.colensoi* (Berk.) G. Cunn. from Australia and New Zealand, *G.gargal* Singer from Argentina and Chile and *G.odorata* Hood, M. Rugolo & Rajchenb. from New Zealand) (Cunningham 1948; Singer 1962; Cunningham 1965; Singer 1969; Buchanan and Ryvarden 2000; Rajchenberg 2006; Rugolo et al. 2022; Rugolo et al. 2023).

*Grifola* species were formerly placed in several different genera, including *Boletus* (Dickson 1785), *Polyporus* (Hooker 1855), *Cautinia* (Maas Geesteranus 1967) and *Hydnum* (Bresadola 1925). In 1821, *Grifola* was erected with the introduction of six new species (Gray 1821), but later, only two species, *G.frondosa* and *G.platypora* Gray, have been accepted (Rugolo et al. 2023), four species have been recombined into different genera as synonyms, *Grifolavaria* (Pers.) Gray as *Cerioporusvarius* (Pers.) Zmitr. & Kovalenko (Zmitrovich and Kovalenko 2016), *Grifolalucida* (Curtis) Gray as *Ganodermalucidum* (Curtis) P. Karst. synonyms (Karsten 1881), *Grifolacristata* (Schaeff.) Gray as *Laeticutiscristata* (Schaeff.) Audet synonyms (Audet 2010), *Grifolabadia* (Pers.) Gray as *Picipesbadius* (Oer.) Zmitr. & Kovalenko synonyms (Zmitrovich and Kovalenko 2016).

*Grifolafrondosa* is an edible mushroom cultivated in different countries, known as “hen of the woods” or “maitake”. It is reported for producing anti-diabetic (n-hexane extract, glycoprotein, and ergosterol peroxide (Konno et al. 2013; Shen et al. 2015; He et al. 2016); anti-tumor (glycoprotein, water soluble extract (Shomori et al. 2009; Cui et al. 2013), anti-virus (protein, Gu et al. 2007) and antioxidant (protein and ergosterol, ergostra-4, 6, 8 (14), 22-tetraen-3-one, and 1-oleoyl-2-linoleoyl-3-palmitoylglycerol (Zhang et al. 2002)) compounds.

Recently, molecular phylogenetic approaches have increasingly been applied to investigate phylogenetic relationships among genera and species of Polyporales (Justo and Hibbett 2011; Justo et al. 2017). Through these studies, *Grifola* is strongly supported as Grifolaceae, with close relationship to the Polyporaceae (Justo and Hibbett 2011; Justo et al. 2017).

For the past 50 years, *Grifola* species have been described based only on morphological characteristics, until the advent of molecular phylogeny. Rugolo et al. (2023) provided molecular markers (ITS and *TUBB*), bringing more evidence for the classification of *Grifola* species.

During investigations on *Grifola* across southwestern China, several *Grifola* collections were made. Amongst them, two *Grifola* species from Yunnan, China, are newly described herein. In addition to the morphological descriptions and illustrations, molecular phylogenetic analyses based on the ITS1-5.8S-ITS2, and *TUBB* supported the two new species.

## ﻿Materials and methods

### ﻿Morphological studies

Macro-morphological characteristics and habitat descriptions were gathered from photographs and field notes. Color codes were assigned according to Kornerup and Wanscher’s (1978). After recording the macromorphological characteristics, specimens were subjected to drying at 40 °C in a food dehydrator until all moisture was eliminated. The dried specimens were then stored in sealed plastic bags. In the microscopic study, dried mushroom materials were sliced and placed in a 5% KOH solution and 1% Congo red for mounting. Microscopic features such as basidia, basidiospores, and cystidia were examined and photographed using a light microscope (Nikon Eclipse 80i) equipped for the purpose. In the descriptions of microscopic characters, measurements were conducted on 60–100 basidiospores and 20 basidia randomly selected. The notation [x/y/z] indicates x basidiospores measured from y basidiomata of z collections. Basidiospore dimensions are denoted as (a–) b–c (–d), where the range b–c represents 95% of the measured values, and “a” and “d” are extreme values. Q refers to the length/width ratio of individual basidiospores, while Q_m_ refers to the average Q value ± standard deviation. Specimens of the two newly discovered *Grifola* species were stored at the herbarium of the Kunming Institute of Botany, Chinese Academy of Sciences (KUN-HKAS).

### ﻿DNA extraction, PCR amplification and sequencing

Genomic DNA extraction from dry specimens was performed using the Ezup Column Fungi Genomic DNA Extraction Kit (Genesand Biotech Co., Ltd, China, Beijing), following the manufacturer’s protocol. Subsequent steps included PCR amplification, purification of PCR products, and sequencing. The primers used for *TUBB* amplification were BTG3F and BTG5G (Shen et al. 2002). The ITS gene region was amplified using the primers ITS4 and ITS5, ITS2 and ITS3 (White and Hedenquist 1990).

### ﻿Sequence alignment and phylogenetic analyses

The sequences of *Grifola* species obtained in this study, along with sequences retrieved from GenBank (refer to Table 1), were aligned using MAFFT version 7 (Katoh and Standley 2013) and verified in BioEdit version 7.0.5 (Hall 2007). Consistent with previous phylogenetic investigations, *Polyporusumbellatus* (Pers.) Fr. and *P.squamosus* P.K. Buchanan & Ryvarden were employed as outgroup taxa (Shen et al. 2002).

**Table 1.** Names, specimen vouchers, origin, and corresponding GenBank accession numbers of the sequences used in this study. New taxa are in bold; “*” following a species name indicates that the specimen is the type of that species and “N/A” refers to the unavailability of data.

Taxon	Voucher specimen	Origin	Host	GenBank accession no.		Reference
				ITS	*TUBB*	
*Grifolacolensoi*	MEL 2320791	Australia	*Eucalyptus*	OP168968	N/A	Rugolo et al. 2023
	MEL 2106744	Australia	*Lophozonia Cunninghamii*	OP168967	N/A	Rugolo et al. 2023
***G.edulis***	HKAS 131996*	**China**	***Lithocarpuscorneus***	**PP079954**	**PP097725**	**This study**
	**HKAS 131997**	**China**	***Lithocarpuscorneus***	**PP079955**	**PP097726**	**This study**
*G.gargal*	CIEFAPcc-700	Argentina	*Lophozoniaobliqua*	OP168980	OP455971	Rugolo et al. 2023
	CIEFAPcc-327	Argentina	*Populusnigra*	OP168991	N/A	Rugolo et al. 2023
	HCFC 3143	Argentina	*Lophozoniaalpina*	OP168989	OP455976	Rugolo et al. 2023
	SGO 092562*	Chile	N/A	N/A	OP455979	Rugolo et al. 2023
*G.odorata*	NZFRIM 1676*	New Zealand	*Podocarpus* sp.	OP168994	N/A	Rugolo et al. 2023
	PDD 86931	New Zealand	*Fuscosporasolandri*	GU222266	OP455985	Rugolo et al. 2023
***G.sinensis***	HKAS 131995*	**China**	***Lithocarpuscorneus***	**PP079956**	**PP097727**	**This study**
	**HKAS 131998**	**China**	***Lithocarpuscorneus***	**PP079957**	**PP097728**	**This study**
	**HKAS 131994**	**China**	***Lithocarpuscorneus***	**PP079958**	**PP097729**	**This study**
*G.sordulenta*	CIEFAPcc-699	Argentina	*Nothofagusdombeyi*	OP168974	N/A	Rugolo et al. 2023
	CIEFAPcc-280	Argentina	*Nothofagusdombeyi*	OP168973	OP455969	Rugolo et al. 2023
*G.frondosa*	WC493	Norway	*Quercusrobur*	AY049128	AY049180	Shen et al. 2002
*Polyporusumbellatus*	Pen13513	China	N/A	KU189772	KU189862	Zhou et al. 2016
*P.squamosus*	Cui 10595	China	N/A	KU189778	KU189868	Zhou et al. 2016

Phylogenies and node support were initially deduced through Maximum Likelihood (ML) using RAxML-HPC2 version 8.2.12 (Stamatakis 2014). This process involved separate analyses of the three single-gene alignments, with 1,000 rapid bootstraps, and was executed on the Cipres portal (Miller et al. 2010). Since there was no identified conflict with substantial support (bootstrap support value (BS) ≥ 70%) among the topologies, the three single-gene alignments were concatenated using SequenceMatrix (Vaidya et al. 2011). For partitioned Maximum Likelihood (ML) the concatenated dataset was analyzed, following the previously mentioned procedure. In the case of Bayesian Inference (BI), the optimal substitution model for each character set was identified using the program MrModeltest 2.3 (Nylander et al. 2004) on the CIPRES platform. The selected models were K80+I for 5.8S, TIM1ef+G for ITS1+ITS2, JC+I+G for *TUBB* extron, F81+G for *TUBB* intron. Bayesian analysis was performed using MrBayes version 3.2.7a (Ronquist et al. 2011) as implemented on the Cipres portal (Miller et al. 2010); two runs of six chains each were conducted by setting generations to 800,000 and stoprul command with the stopval set to 0.01, and trees sampled every 200 generations. A clade was considered to be strongly supported if showing a BS ≥ 70% and a posterior probability (PP) ≥ 0.90. The alignment was submitted to Figshare (10.6084/m9.figshare.24923559).

## ﻿Results

### ﻿Phylogenetic analyses

A total of 10 newly generated sequences and 16 sequences from GenBank were used as ingroup. Four sequences of *Polyporusumbellatus* and *P.squamosus* retrieved from GenBank were used as outgroup. The alignments of the 5.8S, ITS1+ITS2, *TUBB* extron and *TUBB* intron sequences were 158, 396, 404, and 180 characters long after trimming, respectively. The combined data set had an aligned length of 1,138 characters, of which 721 characters were constant, 417 were variable but parsimony-uninformative, and 288 were parsimony-informative.

**Figure 1.**
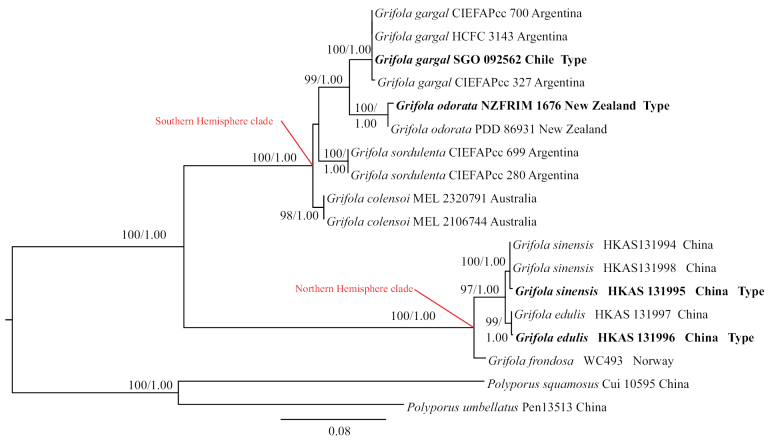
Strict consensus tree illustrating the phylogeny based on the combined 5.8S, ITS1+ITS2, *TUBB* extron and *TUBB* intron data set. Maximum likelihood bootstrap proportions equal to or higher than 70%, and Bayesian posterior probabilities equal to or higher than 0.90 are indicated at nodes. The two *Polyporus* species were used as the outgroup. Holotype specimens are in bold.

ML and BI analyses generated nearly identical tree topologies with little variation in statistical support. Therefore, only the ML tree is displayed (Fig. 1). Phylogenetic data together with thorough morphological analysis (see below) showed that the two newly described taxa in this study are significantly different from other known *Grifola* species.

### ﻿Taxonomy

#### ﻿ Grifola edulis

Taxon classificationFungiPolyporalesGrifolaceae

S.M. Tang & S.H Li sp. nov.

4C2EF63C-66B5-5A14-A197-1CF3970D32B3

MycoBank No: 851587

**Figure 2.**
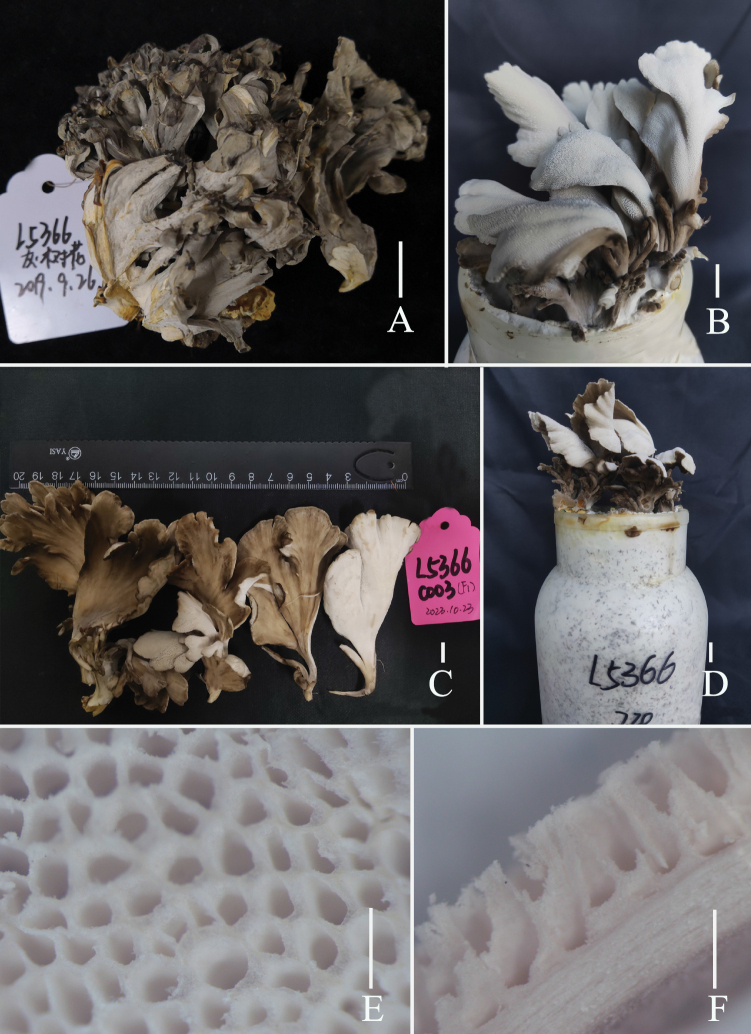
Fresh basidiomata of *Grifolaedulis* (holotype HKAS131996) **A** wild basidiomata **B–D** cultivated basidiomata **E** view of pores by stereoscope **F** side view of pore zone and context by stereoscope. Photographs by Song-Ming Tang. Scale bars: 1 cm (**A–D**); 1 mm (**E, F**).

**Figure 3.**
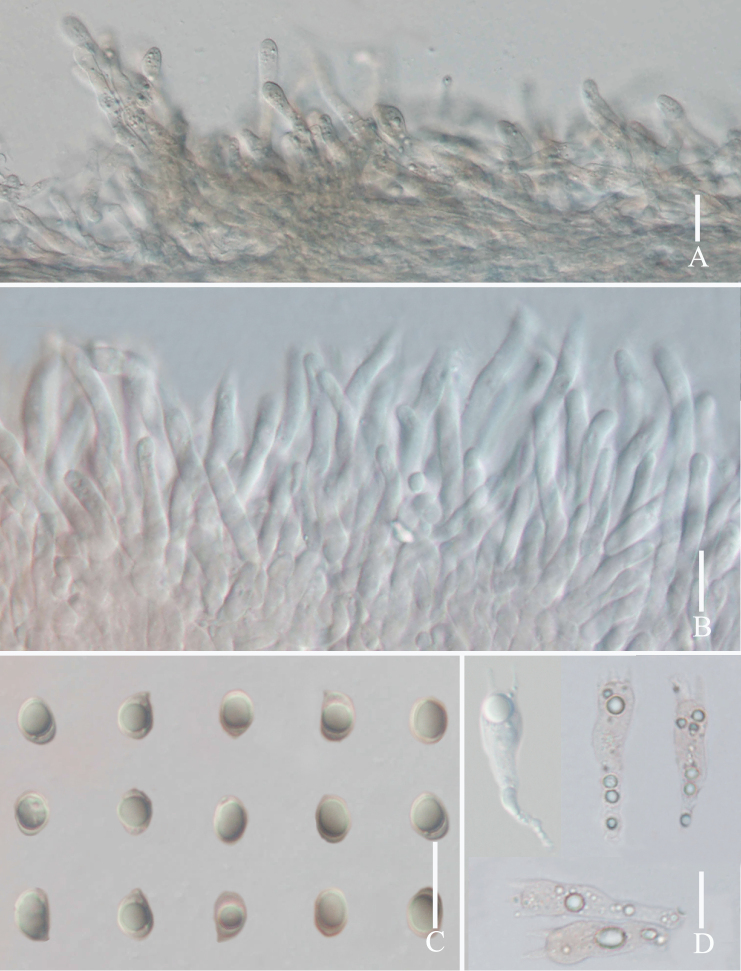
Micromorphological features of *Grifolaedulis* (holotype HKAS131996) **A** cuticle hyphae **B** pore edge **C** basidiospores **D** basidia. Photographs by Song-Ming Tang. Scale bars: 10 μm.

**Figure 4.**
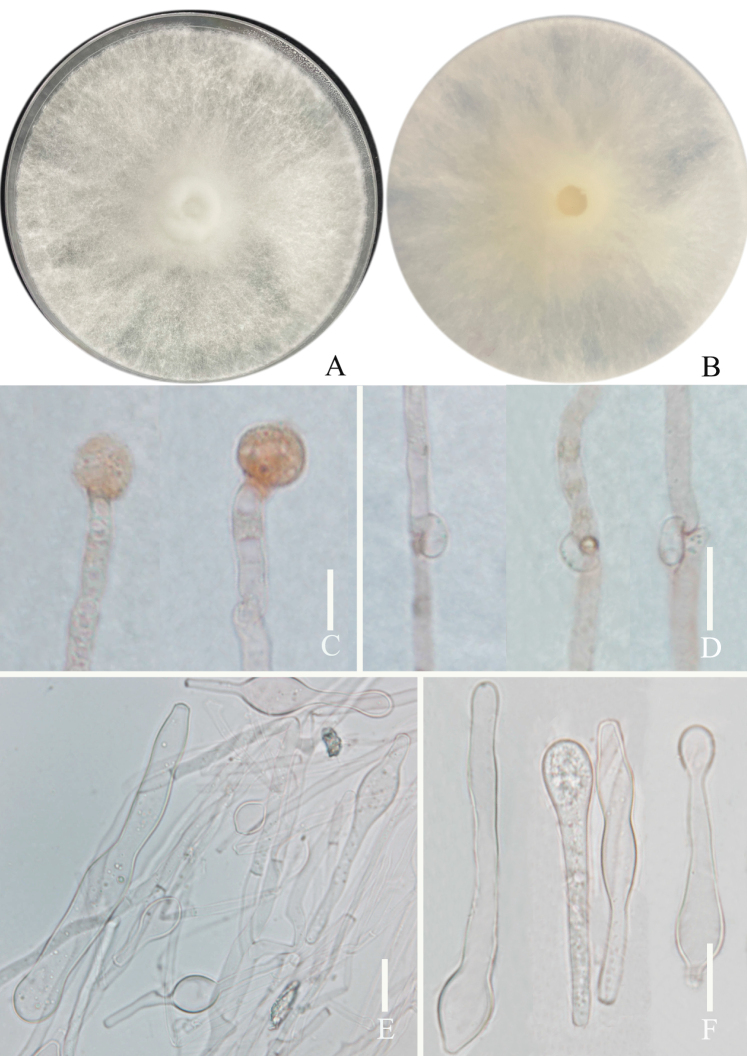
*Grifolaedulis* culture characters (holotype HKAS131996) **A** colony obverse on PDA **B** colony in reverse **C** terminal chlamydospore **D** clamped generative hyphae **E–F** chlamydospores. Photographs by Song-Ming Tang. Scale bars: 10 μm (**C–F**).

Figs 2 , 3 , 4 , 10A, B

##### Etymology.

The epithet “edulis” refers to the edibility of this species, locally considered a delicacy.

##### Holotype.

China. Yunnan province: Nujiang prefecture, Liuku town, elev. 2,300 m, 8 September 2019, Shu-Hong Li, L5366 (**holotype**:HKAS 131996!).

##### Diagnosis.

Differs from other *Grifola* species in having variable and longer chlamydospores (13–) 22–94 (–115) × 7–12 μm, av. 49.8 ± 28.5 × 9.4 ± 1.4 μm, medium-sized basidiomata 12 × 10 × 18 cm, and growing at the base of *Lithocarpuscorneus*.

##### Description.

Basidiomata medium-sized, developing a fruiting structure composed of multiple flattened lobes that emanate from a central base, up to 12 × 10 × 18 cm. Lobes 5–7 cm wide, 8–10 cm long, upper surface gray to gray-brown, lower surface white. Thin cuticle. Context white, 0.5–1 mm. Pores are sizable and often have a convoluted, maze-like appearance, 2–4 per mm, tube layer 2–3 mm deep. Texture fleshy to cartilaginous, becoming hard and woody upon drying, emitting a pronounced almond scent when fresh or dry.

Skeletal hyphae with repent and abundant suberect, thin, aligned parallel longitudinal alone lobe, non-staining in IKI– and 5% NaOH solution, hyphae 5–7 μm wide, terminal slightly enlarged, hyphae 8–10 μm wide. Pores edge heteromorphous, more in number of parallel hyphae, thin-walled, colorless in 5% NaOH solution, 2–4 μm wide; pores trama regular, parallel, 80–120 μm wide, made up of thin-walled, cylindrical hyphae, 2–5 μm wide.

**Figure 5.**
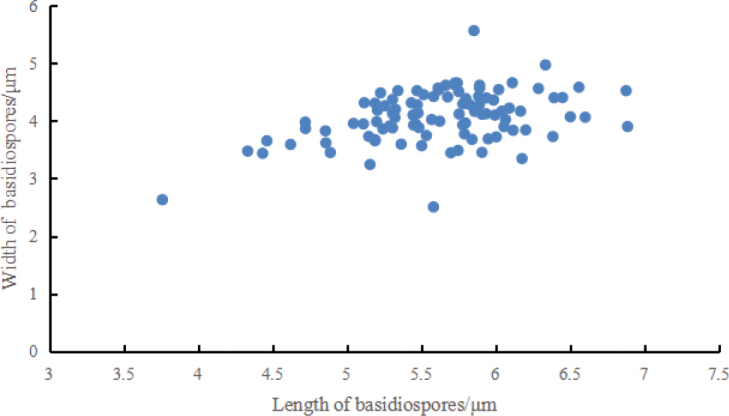
Basidiospores scatter plot of *Grifolaedulis*.

Basidia 17–29 × 5–7 μm, av. 24.6 ± 4.7 × 6.5 ± 0.5 μm, clavate, thin-walled, mostly 4–spored, rarely 2–spored; sterigmata 2–5 μm long. Basidiospores [100/2/2] (3.7–) 4.4–6.8 × 2.5–5.6 μm, av. 5.5 ± 0.5 × 4.1 ± 0.5 μm, Q = 1.1–1.8 (–2.2), Q_m_ = 1.40 ± 0.18, broadly ellipsoid to ellipsoid, colorless in IKI– and 5% NaOH solution, thin-walled, irregular ornamented (Fig. 10); basidiospores scatter plot see Fig. 5.

Culture feature (Fig. 4). Colony regular, circular, greenish gray (1B2) to grayish yellow (1B3); reverse pale yellow (1A3). Dimitic hyphal system, generative hyphae rarely branched. Texture sub felty and farinaceous. Growth slow, 4 cm in 3 weeks, on Potato Dextrose Agar with Chloramphenicol and 24 °C. Mycelium with no distinctive odor, hyphae clamped, thin-walled, and colorless in 5% NaOH solution, 3–6 μm wide. Chlamydospores terminal or intercalary, irregularly, thin-walled, mostly tibiiform or narrowly clavate, rarely narrowly lageniform or ellipsoid, (13–) 22–94 (–115) × 7–12 μm, av. 49.8 ± 28.5 × 9.4 ± 1.4 μm, Q = 1.4–8.3 (–15.9), Q_m_ = 5.4 ± 3.5, colorless in 5% NaOH solution.

##### Habitat and distribution.

*Grifolaedulis* occurs in native forests in Yunnan, on *Lithocarpuscorneus* at the base of trees, producing an aromatic white rot.

##### Edibility.

This mushroom is highly appreciated by local communities.

##### Additional material examined.

China. Yunnan province: Lushui city, Laowo town, altitude 1,755 m, 12 August 2020, Shu-Hong Li, HKAS 131997.

##### Notes.

*Grifolaedulis* is close to *G.frondosa* and *G.amazonica*, until now the only species that have been described and recorded from the Northern Hemisphere (Shen et al. 2002; Rugolo et al. 2023). However, in *G.frondosa*, lobes’ upper surface is gray to brown tomentose, basidiospores 5.5–6.5 × 3.5–4.5 μm, fruiting bodies occur from September to October, growing on *Quercus*, *Castanea*, *Fagus*, and *Carpinus* (Rugolo et al. 2023); *G.edulis* presents lobes’ upper surface gray to gray brown, smooth, smaller basidiospores av. 5.5 ± 0.5 × 4.1 ± 0.5 μm, and fruiting bodies occur from August to September, on *Lithocarpuscorneus*. *Grifolaamazonica* from Brazil, has lobes’ upper surface evenly brown, glabrous to smooth, smaller basidiospores 4–4.5 × 3–3.5 μm, and pore surface pale grayish brown (Ryvarden 2004).

In our multi-locus phylogeny, *G.frondosa* and *G.sinensis* are sister to the clade of *G.edulis*. Specimen WC493 (from Norway) has the representative sequence for *G.frondosa*, given the original collection of *G.frondosa* in Europe (Britain). The *TUBB* genetic distances between *G.edulis* (holotype HKAS 131996) and other accessions in the latter clade were 4.50% (26/578) for *Grifolafrondosa* (WC493), 1.21% (7/578) for *G.sinensis* (holotype HKAS 131995), thus classifying them as heterospecific.

#### ﻿ Grifola sinensis

Taxon classificationFungiPolyporalesGrifolaceae

S.M. Tang & S.H. Li sp. nov.

F5F19E9E-2D42-5BFA-8C3E-65C117537593

MycoBank No: 851588

**Figure 6.**
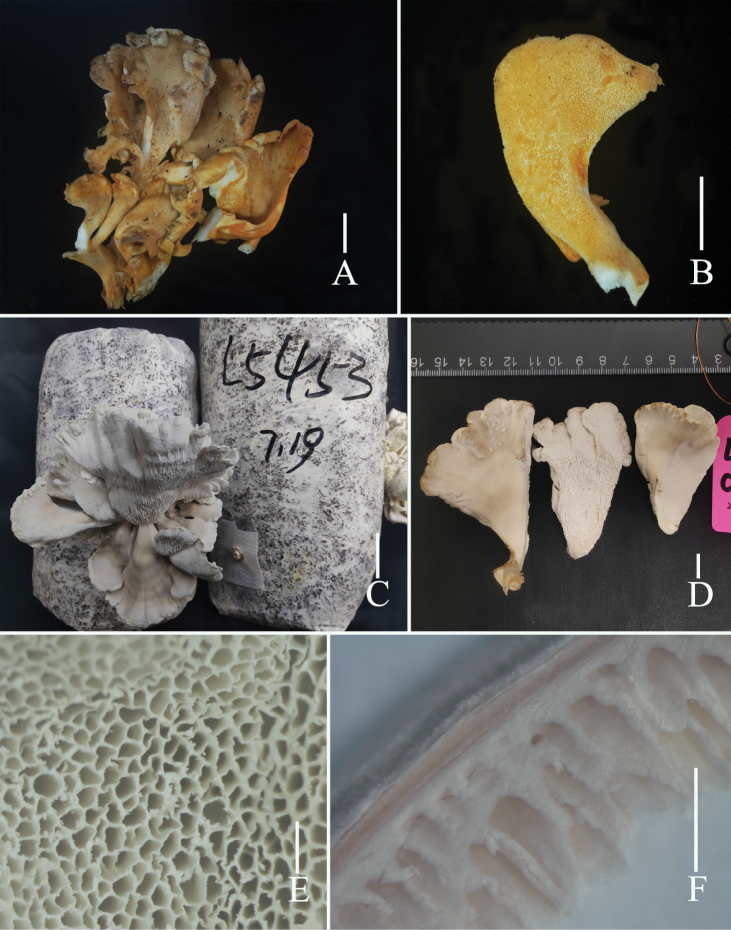
Fresh basidiomata of *Grifolasinensis* (holotype HKAS 131995) **A** view of wild basidiomata pilei **B** view of wild basidiomata pores **C, D** cultivated basidiomata **E** view of pores by stereoscope **F** side view of pore zone and context by stereoscope. Photographs by Song-Ming Tang. Scale bars: 1 cm (**A–D**); 1 mm (**E, F**).

**Figure 7.**
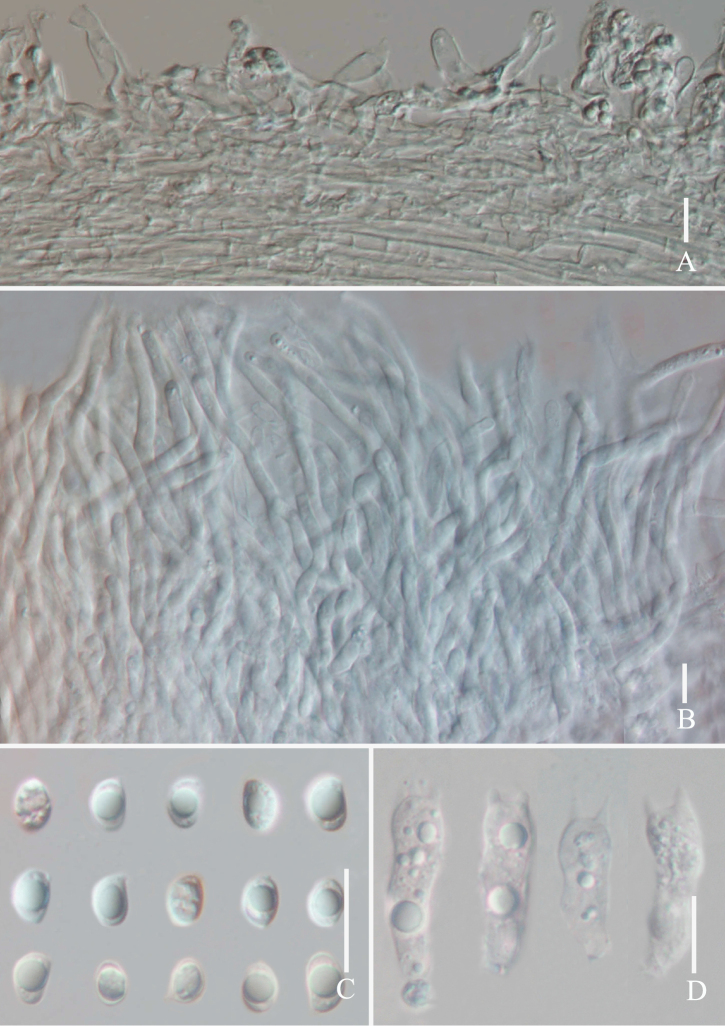
Micromorphological features of *Grifolasinensis* (holotype HKAS 131995) **A** cuticle hyphae **B** pore edge **C** basidiospores **D** basidia. Photographs by Song-Ming Tang. Scale bars: 10 μm.

**Figure 8.**
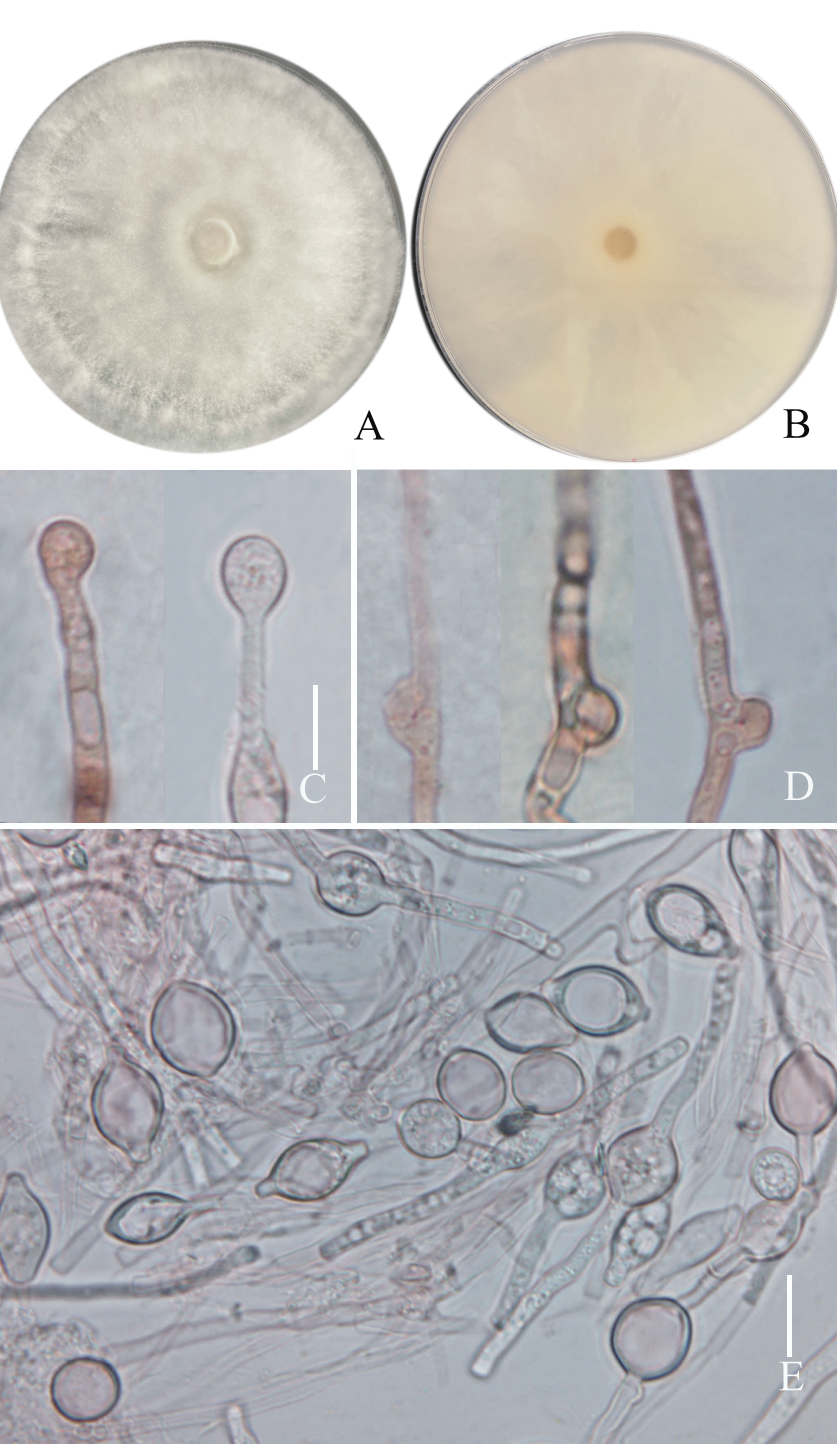
*Grifolasinensis* cultures characters (holotype HKAS 131995) **A** colony obverse on PDA **B** colony in reverse **C** terminal chlamydospore **D** clamped generative hyphae **E, F** chlamydospores. Photographs by Song-Ming Tang. Scale bars: 10 μm (**C–F**).

Figs 6 , 7 , 8 , 10C, D

##### Etymology.

The epithet “sinensis” refers to the country China where this fungus was first discovered.

##### Holotype.

China. Yunnan province: Nujiang prefecture, Fugong city, elev. 2,230 m, 8 September 2019, Shu-Hong Li, L5453 (**holotype**: HKAS 131995!).

##### Diagnosis.

Differs from other *Grifola* species in having a medium-sized basidiomata, with white to olive yellow lobes, smaller and irregular pore (2–4/mm), and ellipsoid to narrowly utriform chlamydospores.

##### Description.

Basidiomata medium-sized, developing a fruiting structure composed of multiple flattened lobes that emanate from a central base, up to 10 × 12 × 15 cm. Lobes 4–7 cm wide, 7–10 cm long, lower and upper surface white (1A1) to grayish white (1A2) when young, changing to olive yellow (2C–D7) with age or when soaked. Thin cuticle. Context white, 1–2 mm thick. Pores often with a convoluted, maze-like appearance, 2–4 per mm, tubes 2–3 mm deep. Texture fleshy to cartilaginous, becoming hard and woody upon drying, and emitting a pronounced almond scent when fresh or dry.

Skeletal hyphae aligned parallel longitudinal alone lobe, with repent and abundant suberect terminal segments, hyphae thin-walled, non-staining in IKI and 5% NaOH solution, 5–7 μm wide. Pores edge heteromorphous, hyphae thin-walled, colorless in 5% NaOH solution, 2–4 μm wide; trama of tubes regular, parallel, 120–190 μm wide, made up of thin-walled hyphae, 2–5 μm wide.

**Figure 9.**
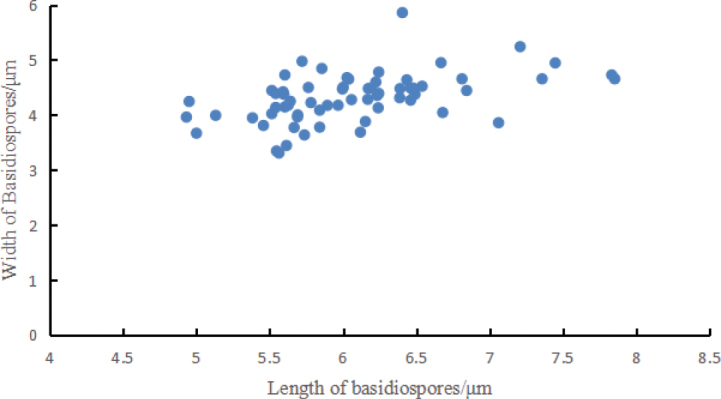
Basidiospores scatter plot of *Grifolasinensis*.

**Figure 10.**
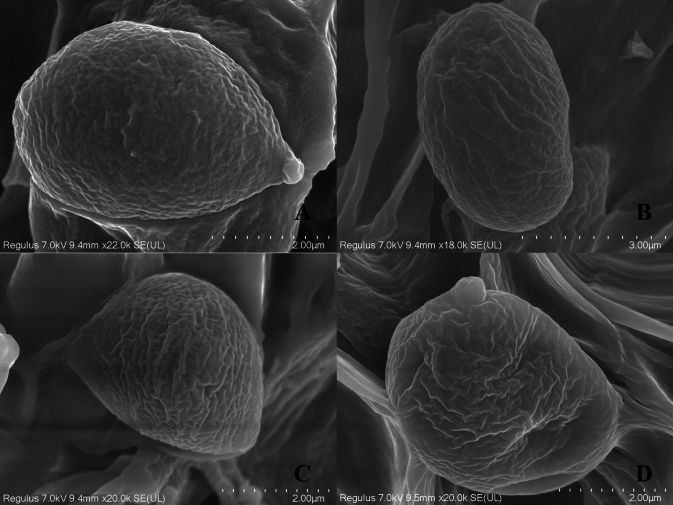
Characteristics of basidiospores ornamentations **A, B***Grifolaedulis* (HKAS 131996) **C, D***Grifolasinensis* (HKAS 131995).

Basidia 15–28 (–32) × 5–8 μm, av. 23.0 ± 5.4 × 6.7 ± 0.7 μm, clavate, thin-walled, mostly 2–spored, rarely 4–spored; sterigmata 2–5 μm long. Basidiospores [68/2/2] 4.6–7.9 × 3.0–5.9 μm, av. 5.9 ± 0.6 × 4.2 ± 0.5 μm, Q = 1.1–1.6 (–1.8), Q_m_ = 1.42 ± 0.15, broadly ellipsoid to ellipsoid, colorless in IKI and 5% NaOH solution, thin-walled, irregular ornamented (Fig. 10); basidiospores scatter plot, see Fig. 9.

Culture feature (Fig. 8). Colony regular, circular, greenish gray (1B2) to grayish yellow (1B3); reverse pale yellow (1A3). Dimitic hyphal system, generative hyphae rarely branched. Texture sub felty and farinaceous. Growth slow, 4 cm in 3 weeks on Potato Dextrose Agar with Chloramphenicol and 24 °C. Mycelium with no distinctive odor, generative hyphae clamped, thin-walled, and colorless in 5% NaOH solution, 3–5 μm wide. Presence of chlamydospores terminal or intercalary, mostly ellipsoid, rarely narrowly utriform, 9.6–16.1 (–21.9) × 7.4–11.9 μm, av. 13.4 ± 2.9 × 9.2 ± 1.2 μm, Q = 1.1–2.0 (–2.9), Q_m_ = 1.5 ± 0.5, colorless in 5% NaOH solution, thin-walled. Generative hyphae hyaline, thin walled, clamped, 2.7–4.3 μm, av 3.6 ± 0.6 μm, hyphal endings arranged singly or in groups, with contents stained red in Congo solution.

##### Habitat and distribution.

*Grifolasinensis* occurs in native forests in Yunnan, on *Lithocarpuscorneus*, at the base of trees, causing an aromatic white rot.

##### Edibility.

This species is much appreciated by the locals in Yunnan, stir-frying it over high heat with green peppers; it has a robust almond essence that permeates through the palate, accompanied by a hearty, meat-like texture.

##### Additional species examined.

China. Yunnan Province, Nujiang prefecture, Fugong city, elev. 2,120 m, 5 September 2019, Shu-Hong Li, HKAS 131998; Nujiang prefecture, Bingzhongluo county, elev. 1,980 m 15 October 2023, Song-Ming Tang, HKAS 131994.

##### Notes.

Morphologically, *G.sinensis* is similar to *G.amazonica* Ryvarden in having small irregular pores 2–4/mm. However, *G.amazonica* has evenly brown lobes, smaller basidiospores 4–4.5 × 3–3.5 μm, and basidia 12–14 × 3.5–4.5 μm, grows on dead hardwood trees, and its distribution is in the North Hemisphere (Ryvarden 2004).

*Grifolagargal* Singer is close to *G.sinensis*, both having cream yellow pilei, and pores 1–2/mm. However, *G.gargal* has larger basidiospores, 7–8 × 5–6 μm, and monomitic hyphal system (Singer 1969; Rugolo et al. 2023).

In our multi-locus phylogeny, *G.sinensis* is closely related to *G.frondosa* and *G.edulis*. However, *G.frondosa* has dark to pale gray pilei, larger basidiomata, up to 40–50 cm, and white pores. *Grifolaedulis* has irregular, mostly tibiiform or narrowly clavate, rarely narrowly lageniform or ellipsoid and relatively larger chlamydospores, (13–) 22–94 (–115) × 7–12 μm, av. 49.8 ± 28.5 × 9.4 ± 1.4 μm, gray to gray-brown pilei and cuticle hyphae terminal segments slightly enlarged (this study).

## ﻿Discussion

In this study, we combined sequences of four non-translated loci (5.8S, ITS1+ITS2, *TUBB* extron and *TUBB* intron) to carry out phylogenetic analyses of *Grifola* species, in order to investigate the phylogenetic relationships between the two new species we described and other *Grifola* species. At present, eight *Grifola* species have been described in the world, including this study two novel species, each species are given in the Table 2.

**Table 2.** Synopsis of the species of *Grifola*.

Species	Basidiospores	Basidia	Pilei surface	Pores	Chlamydospores	Basidiomata size and hyphal system	Host	Reference
*G.amazonica*	Ellipsoid; 4–4.5 × 3–3.5 μm	12–14 × 3.5–4.5 μm	Deep purplish bay to dark brown	Pore surface pale grayish brown; pores 3–5 per mm; tubes concolorous, 5 mm deep	–	Up to 8 cm wide; dimitic hyphal system	On dead hardwood tree	Ryvarden L. 2004
*G.colensoi*	4–5 × 4–5 μm	–	Smoky brown, dark brown or purplish black	Pores large, irregular, usually rather elongated laterally, radially arranged	–	32 × 27 × 25 cm; dimitic hyphal system	*Fuscosporafusca* and *Eucalyptus*	Cunningham 1965; Rugolo et al. 2023
***G.edulis***	**(3.7–) 4.4–6.8 × 2.5–5.6 μm; av. 5.5 ± 0.5 × 4.1 ± 0.5 μm**	**17–29 × 5–7 μm**	**Gray to gray-brown**	**Pore surface white; tubes 2–3 mm deep; pores 2–4 per mm**	**Mostly tibiiform or narrowly clavate, rarely narrowly lageniform or ellipsoid, (13–) 22–94 (–115) × 7–12 μm**	**12 × 10 × 18 cm; dimitic hyphal system**	***Lithocarpuscorneus***	**This study**
*G.gargal*	Ellipsoid; 7–8×5–6 μm	–	Cream yellow, light brown or gray	Pore surface white; tubes up to 5 mm deep; pores 1–2 per mm	–	Up to 30 cm wide; monomitic hyphal system	*Lophozoniaobliqua*, *L.alpina*, *Weinmania*, *Amomyrtus*, and *Eucryphia*	Singer 1969; Rajchenberg 2002, 2006
*G.odorata*	Subglobose to broadly ellipsoid, 5.8–8.5 × 5–7 μm	30 × 8 μm	Gray, brown, light brown, or white	Pore surface white; pores 1–2 per mm	Subglobose, 10–11 × 7–8 μm	35 × 22 × 24 cm; monomitic hyphal system	*Metrosiderosrobusta*, *M.excelsa*, *Fuscosporasolandri*, and *F.fusca*	Rugolo et al. 2023
***G.sinensis***	**4.6–7.9 × 3.0–5.9 μm, av. 5.9 ± 0.6 × 4.2 ± 0.5 μm**	**15–28 (–32) × 5–8 μm**	**White to grayish white when young, changing to olive yellow with age or when soaked**	**Pore surface white to grayish white when young, changing to olive yellow with age or when soaked; tubes 2–3 mm deep, pores 2–4 per mm**	**Mostly ellipsoid, rarely narrowly utriform, 9.6–16.1 (–21.9) × 7.4–11.9 μm**	**10 × 12 × 15 cm; dimitic hyphal system**	***Lithocarpuscorneus***	**This study**
*G.sordulenta*	Ellipsoid to ovoid; 6–7×4–5 μm	–	Cream color, light cinnamon or grayish	Pore surface cream-color; pores 1–2 per mm	–	35 × 15 × 30 cm; monomitic hyphal system	*Nothofagusdombeyi*	Singer 1969; Rajchenberg 2002, 2006
*G.frondosa*	5.5–6.5 (–7) × 3.5–4.5 μm	–	Pale gray	Pore surface white; pores 2–4 per mm	–	Up to 40–50 cm wide; dimitic hyphal system	*Quercus*, *Castanea*, *Fagus* and *Carpinus*	Gray 1821; Rugolo et al. 2023

Chlamydospores size and shape are important characters for identifying species of *Grifola*, but ignored in previous studies, being Rugolo et al. (2023) the first to provide the description of *G.odorata* chlamydospores. Chlamydospores of *G.edulis* and *G.sinensis* clearly differ in size and shape, in *G.edulis* chlamydospores are irregular, mostly tibiiform or narrowly clavate, rarely narrowly lageniform or ellipsoid, (13–) 22–94 (–115) × 7–12 μm, av. 49.8 ± 28.5 × 9.4 ± 1.4 μm; in *G.sinensis* chlamydospores are mostly ellipsoid, rarely narrowly utriform, 9.6–16.1 (–21.9) × 7.4–11.9 μm, av. 13.4 ± 2.9 × 9.2 ± 1.2 μm, Q = 1.1–2.0 (–2.9).

The phylogenetic analysis conducted by Rugolo et al. (2023) revealed that *Grifola* taxa form two well clades, one from the northern Hemispheres and another from the southern Hemisphere; our research also confirms this result. The North Hemisphere clade includes *G.frondosa*, as well as our collections of *G.edulis* and *G.sinensis*. Shen et al. (2002) studied the isolation of *G.frondosa* worldwide, and identified partition in different phylogenetic species. In this study, we designated specimen WC493 (from Norway) as *G.frondosa*, following the type specimen from Europe (Shen et al. 2002), and the three species are clearly separated in our phylogenetic tree (Fig. 1). The south Hemisphere clade comprises *G.sordulenta*, *G.colensoi*, *G.gargal* and *G.odorata*; species of *G.sordulenta* and *G.colensoi* form a sister clade, are characterized by dark brown or purplish black pilei, with no distinct odor (Rajchenberg 2006).

Previously, Asian *Grifola* isolates were all considered as of *G.frondosa* (Shen et al. 2002). Studies only based on morphology or molecular analyses were insufficiently informative. Combining morphological and phylogenetic analysis, we introduce two new species from Asia, very close to *G.frondosa* (WC493) (Fig. 1). Approximately four decades ago, maitake mushrooms were exclusively sourced from their natural habitat. *Grifolafrondosa* commercial cultivation commenced in Japan, as documented by Takama et al. (1981). Since that time, Japan has emerged as the predominant global producer of maitake, contributing to 98% of the total worldwide production (Chang 1999). Subsequently, industrial cultivation of maitake in China also rapidly developed. In 2022, the annual maitake production in China reached approximately 50,000 tons (from the China Edible Fungi Association). As *Grifolafrondosa*, *G.edulis* and *G.sinensis* form a clade, this implies that *G.edulis* and *G.sinensis* may also have potential cultivation value.

Species of *Grifola* host are variable, including genera *Eucalyptus*, *Lophozonia*, *Lithocarpus*, *Populus*, *Podocarpus*, *Fuscospora*, *Nothofagus*, and *Quercus*, most *Grifola* species have different hosts, rarely *Grifola* species only found under the same host (Cunningham 1948; Singer 1962; Cunningham 1965; Singer 1969; Buchanan and Ryvarden 2000; Rajchenberg 2006; Rugolo et al. 2023).

We use 750 mL plastic bottles to cultivate *G.edulis* and *G.sinensis* at room temperature of 20 °C–25 °C and air humidity of 70%–85%; the cultivated material is 80% sawdust, 18% wheat bran, 1% sugar and 1% gypsum, the biological conversion rate of *G.edulis* and *G.sinensis* is approximately 20%.

*Grifolaedulis* and *G.sinensis* are widely distributed in the subtropical broad-leaved forests of Gongshan city in Yunnan, where the annual average temperature is 11–22 °C, and the elevation is between 1,170–5,128 m (Wang 2018). In China, Yunnan, there is a tropical to subtropical climate suitable for abundance of fungal resources, so certainly more *Grifola* species will be discovered in the future.

## Supplementary Material

XML Treatment for Grifola edulis

XML Treatment for Grifola sinensis

## ﻿Additional information

### Author ORCIDs

Song-Ming Tang https://orcid.org/0000-0002-6174-7314

De-Chao Chen https://orcid.org/0009-0006-3626-1191

Shuai Wang https://orcid.org/0009-0001-0157-6859

Xiao-Qu Wu https://orcid.org/0009-0006-7980-2322

Cheng-Ce Ao https://orcid.org/0000-0002-1569-3386

Er-Xian Li https://orcid.org/0009-0008-4671-8698

Hong-Mei Luo https://orcid.org/0009-0001-9976-4371

Shu-Hong Li https://orcid.org/0000-0001-5806-9148

### Data availability

All of the data that support the findings of this study are available in the main text.
